# New Insights of Scaffolds Based on Hydrogels in Tissue Engineering

**DOI:** 10.3390/polym14040799

**Published:** 2022-02-18

**Authors:** Denisa-Maria Radulescu, Ionela Andreea Neacsu, Alexandru-Mihai Grumezescu, Ecaterina Andronescu

**Affiliations:** 1 Department of Science and Engineering of Oxide Materials and Nanomaterials, Faculty of Applied Chemistry and Materials Science, University Politehnica of Bucharest, 011061 Bucharest, Romania; denisa.m.radulescu@gmail.com (D.-M.R.); grumezescu@yahoo.com (A.-M.G.); ecaterina.andronescu@upb.ro (E.A.); 2 Academy of Romanian Scientists, 54 Independentei, 050094 Bucharest, Romania; 3 National Research Center for Micro and Nanomaterials, Faculty of Applied Chemistry and Materials Science, University Politehnica of Bucharest, 060042 Bucharest, Romania; 4 Research Institute of the University of Bucharest (ICUB), University of Bucharest, 050657 Bucharest, Romania

**Keywords:** hydrogels, scaffolds, polymers, tissue engineering, healing process, regeneration

## Abstract

In recent years, biomaterials development and characterization for new applications in regenerative medicine or controlled release represent one of the biggest challenges. Tissue engineering is one of the most intensively studied domain where hydrogels are considered optimum applications in the biomedical field. The delicate nature of hydrogels and their low mechanical strength limit their exploitation in tissue engineering. Hence, developing new, stronger, and more stable hydrogels with increased biocompatibility, is essential. However, both natural and synthetic polymers possess many limitations. Hydrogels based on natural polymers offer particularly high biocompatibility and biodegradability, low immunogenicity, excellent cytocompatibility, variable, and controllable solubility. At the same time, they have poor mechanical properties, high production costs, and low reproducibility. Synthetic polymers come to their aid through superior mechanical strength, high reproducibility, reduced costs, and the ability to regulate their composition to improve processes such as hydrolysis or biodegradation over variable periods. The development of hydrogels based on mixtures of synthetic and natural polymers can lead to the optimization of their properties to obtain ideal scaffolds. Also, incorporating different nanoparticles can improve the hydrogel’s stability and obtain several biological effects. In this regard, essential oils and drug molecules facilitate the desired biological effect or even produce a synergistic effect. This study’s main purpose is to establish the main properties needed to develop sustainable polymeric scaffolds. These scaffolds can be applied in tissue engineering to improve the tissue regeneration process without producing other side effects to the environment.

## 1. Introduction

Tissue engineering is a multidisciplinary field that involves cells, biomaterials, the interactions between cells and materials, and their characterization through different surface techniques. Their combination leads to enhanced treatment methods and, therefore, the regeneration of new tissues [1,2]. In recent years, the need to obtain tissues and organs for transplantation, regeneration, or the replacement of damaged tissues has become significantly more important compared to the availability of transplanted organs. To this end, functional substitutes can be developed by using a combination of biomaterials, bioactive support molecules, or cells, followed by in vitro culture or in vivo implantation. [3].

Even though tissue engineering has been considered a promising strategy, one of the most important obstacles to its progress is represented by the lack of specific materials for scaffold development [4]. In this direction, scaffolds are known as biomaterials synthesized by using various compounds that allow cells seeding into their system and the attachment of active principles to the inner walls of the cavities. Hence, hydrogels can provide adequate mechanical strength, thus improving the network’s biological activity by mimicking the extracellular matrix (ECM) [5,6,7]. For example, hydrogels are among the main used materials to overcome this deficiency due to their ability to mimic many features of the native cellular microenvironment, allowing their application in tissue engineering and regenerative medicine [8,9].

The polymer matrix ensures proliferation, adhesion, and cell migration capacity. These are essential characteristics necessary for functional tissue development [10]. Due to their mechanical and chemical properties similar to those of native tissues, hydrogels are promising materials that can be used as scaffolds for this domain [11]. Recently, numerous classes of hydrogels have been developed for the incorporation of the cell, drugs, or bioactive molecules. The developed system can respond specifically to certain types of stimuli from the internal or external environment of cells and tissues [12]. Therefore, hydrogels can be considered the most suitable material used in applications, such as wound healing, cell therapy, soft or hard tissue engineering, and controlled drug release [13]. Hence, this study’s main purpose is to establish the necessary properties for the development of sustainable polymeric scaffolds, which can be further used in tissue engineering to improve the regeneration process without producing other adverse effects on the environment.

## 2. Need and Significance of Hydrogel-Based Scaffold

Over the years, it was observed that scaffolds have great importance for tissue engineering applications as they should be able to guide cell growth, found in their structure, and at the same time, aid the cell migration process from surrounding tissues. The crucial characteristics of these biomaterials must provide the essential chemical and physical properties for cell attachment promotion, migration, proliferation, and differentiation. Even though, the already developed and commercialized scaffolds provide unsatisfactory interaction with stromal cells and inadequate cell mimicking ability that is necessary for tissue regeneration. A suitable alternative to overcome these limitations is represented by hydrogel-based scaffolds. Their 3D and hydrophilic structure render them the ability to maintain considerable amounts of water or other biological fluids [14,15]. The polymeric scaffolds are composed of dynamic crosslinking structures that do not affect their integrity. Therefore, the high-water content found in hydrogels aids nutrient diffusion. Moreover, their substantial flexibility and elasticity [16], similar to the native ECM, provide the necessary biochemical and structural support to surrounding cells and influence the fate of tissue formation. Further, increasing the efficacy of hydrogel-based scaffolds for clinical applications became the main priority. Considering this, their performance has been demonstrated by evaluating the necessary mechanical, biological, and clinical requirements. A proper hydrogel must be able to regenerate specific tissues, with minimum requirements related to vascularization, cell growth, proliferation, integration, and simultaneous degradation during the healing process. [17]. The created environment qualifies hydrogel-based scaffolds as ideal matrices in which various cells can be cultured to create in vitro tissues [18].

Even though, there are still many concerns related to hydrogels integration and their behavior in contact with the native tissue as the organism exhibits constant modifications. Thus, the need to obtain novel scaffolds for tissue regeneration represents a continuous necessity.

To develop these systems, both natural and synthetic polymers are the main used materials for obtaining hydrogels, as they can interact non-covalently or even be subjected to a crosslinking process, forming an insoluble system [19]. The most important properties of a hydrogel-based scaffold are represented by its biocompatibility and non-toxicity. They also possess superior physical and mechanical stability, high biodegradability, without the appearance of toxic species after the degradation of the polymeric system, high durability, superior absorption capacity in their swollen state [20,21]. By applying stimuli such as light, pH, temperature, or magnetic field, hydrogels can release various active principles that facilitate the healing process [22].

**Figure 1:**
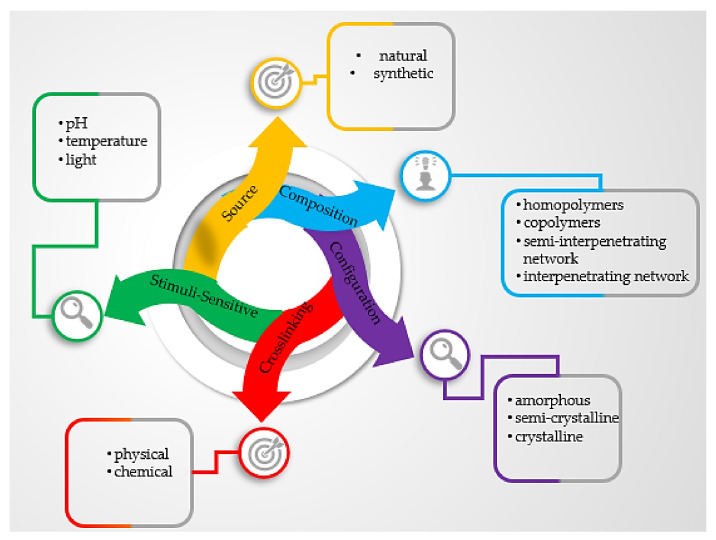
The main classes of hydrogels.

The classification of hydrogels could also influence the biomaterial selection for its targeted application. They are classified based on source, their ionic charges, polymerization process, physical properties, provenience source, triggers, or cross-linkers [23,24], as shown in Figure 1. The polymeric chain networks that form stimulus-sensitive hydrogels can be controlled by physical (e.g., light, temperature, electric fields, mechanical forces) and chemicals stimuli (e.g., biomolecules, ions), leading to a conformational change of polymer chains or variations in polymeric networks (e.g., low or high crosslink density) [25,26,27,28,29].

## 3. Types of Hydrogels Used for Scaffold Development

Following continuous research considering hydrogel-based scaffolds, various formulations have been developed as 3D matrices for their application in tissue engineering. Depending on the provenience of the material, hydrogels can be categorized into natural, synthetic, or hybrid, as shown in Table 1. Hydrogels developed from natural sources represent an ideal candidate due to their increased biocompatibility but are often limited due to their poor stability and mechanical properties [30,31].

Alginate is a commonly used linear and hydrophilic polysaccharide for tissue engineering applications. This polysaccharide is composed of (1–4)-linked β-D-mannuronic acid (M) and α-L-guluronic acid (G) monomers [62]. Over the years, alginate has been widely used for hydrogels development as the gelation process occurs under mild conditions and provides decreased toxicity, good biocompatibility, cost-effectiveness, and broader availability. However, alginate may not be the most suitable polymer as it does not specifically degrade. Tan et al. concluded that ionically crosslinked hydrogels degrade via ion exchange processes which involve divalent ions loss into the surrounding environment that causes an uncontrolled dissolution [63].

Another type of linear and polycationic polysaccharide used for tissue engineering applications is represented by chitosan (CS). CS is known as a partially deacetylated derivative of chitin and contains N-acetylglucosamine and glucosamine molecules. Therefore, the structure of CS is similar to the native glycosaminoglycans. Further, Tan et al. concluded that CS is widely used for hydrogel synthesis for its biocompatibility, excellent biodegradability, and low immunogenicity. Additionally, water-soluble CS derivates support cell growth and can be easily modified via primary amine groups [63].

The most widely used natural polymer is represented by hyaluronic acid (HA), a non-sulfated glycosaminoglycan distributed throughout the ECM of all connective tissues. HA play a crucial role in various biological processes such as proteoglycan organization, tissue hydration, nutrient diffusion, and cell differentiation. The main features of HA are the increased biocompatibility, excellent gel-forming properties, and high biodegradability that recommend them for hydrogels applications. The study performed by Borzacchiello et al. highlighted the successful development of HA hydrogels with increased biocompatibility and high biodegradability for skin tissue applications [64]. Collagen (COLL) hydrogels have been widely researched in tissue engineering due to their weak immunogenicity and excellent biocompatibility. However, all mentioned natural polymers are limited by their weak mechanical properties. This drawback prevents their application in the medical field and therefore, the necessity to combine them with other organic/inorganic molecules to obtain the proper reinforcement remains [63]. In this regard, Agban et al. synthetized crosslinked collagen hydrogels which contained ZnO NPs. The hardness, adhesiveness, and rheological properties of collagen hydrogels are correlated to the added NPs concentration, to improve the mechanical feature of the material [65]. On the other hand, synthetic hydrogels can also be suitable for biomedical applications as their mechanical or physicochemical properties are reproducible and adjustable [66]. Their main disadvantage is the decreased or lack of biocompatibility.

In this regard, polyvinyl alcohol (PVA) is a common polymer used in tissue engineering and provides good solubility, biocompatibility, excellent mechanical properties, non-toxicity, and non-carcinogenicity. PVA is obtained through the full or partial hydrolysis of poly(vinyl acetate). Even though, Pan et al. highlighted that pure PVA hydrogels are not able to provide the desired effects of hemostasis or antibacterial activity. They show a lack of hydrophilicity or elasticity. Over the years, researchers have focused their studies on the combination of PVA with other natural polymers to promote wound healing and improve their biological properties [67].

Furthermore, polyethylene glycol (PEG) hydrogels have been widely selected for wound dressing applications due to their favorable features such as good biocompatibility, biodegradability, non-toxicity, stable activity, easy availability, and low preparation cost. Nevertheless, the used crosslinkers for their synthesis are cytotoxic. Hence, recent studies have been focused on decreasing the toxicity caused by crosslinking agents, focusing on their replacement with more natural compounds such as citric acid (CA) [68].

Another good example of synthetic hydrogels is represented by poly (N-isopropylacrylamide) (pNIPAM). Its structure presents both hydrophobic isopropyl groups and hydrophilic amido groups on the macromolecular chain. The developed hydrogels exhibit temperature-sensitive features that could be considered an advantage for future applications, providing a thermal response. Additionally, pNIPAM has good biocompatibility and biodegradability, non-toxicity, and exhibits physiological reactivity. The developed hydrogels have been extensively used for drug delivery and wound dressing [68].

Poly(2-hydroxyethyl methacrylate) (pHEMA) could also act as a suitable candidate for hydrogels synthesis. pHEMA hydrogels have been studied for many years in the biomedical field for their superior properties such as non-biodegradability, biocompatibility, and optically transparent hydrophilic nature. The swelling ability of the polymer is produced by the hydrophilic pendant group. In his swollen state, pHEMA becomes soft, flexible and allows oxygen and liquid diffusion. Additionally, the synthetic scaffold exhibits superior hydrophilicity, low friction coefficient, and increased cytocompatibility [69,70,71,72]. Furthermore, the acrylate version of this polymer, poly(2-hydroxyethyl acrylate) (PHEA), could be also a suitable constituent for hydrogel development. PHEA polymerization occurs in the presence of ethanol and water. Serrano Aroca et al. provided the successful development of hydrogels through the co-polymerization of ethylene glycol dimethacrylate and of 2-hydroxyethyl acrylate in a solution of methanol [73]. Another study of this group reported that plasma polymerization of poly(2-hydroxyethyl acrylate) hydrogel can increase the mechanical properties of hydrogels, features that make them ideal candidates for hard tissue engineering [74]. Other studies performed by Hernandez et al. performed the successful development of porous acrylate-based scaffolds to substitute partial fibrous rings of human intervertebral disks offering therefore improved mechanical properties and high water uptake [75].

Hybrid or semi-synthetic hydrogels are usually preferred because they combine the advantages of both synthetic and natural polymers. The hydrogel obtained by mixing natural proteins or polymers with synthetic polymeric materials provides biological activity and good mechanical properties. For example, Berkovitch et al. reported the successful hydrogel development based on proteins and polyethylene glycol for encapsulation of dorsal root ganglion cells [30]. Besides, synthetic polymers have tunable chemical and physical properties, with higher reproducibility than natural hydrogels.

## 4. Physical to Chemical Methods Used for Hydrogels Scaffolds Design

**Figure 2:**
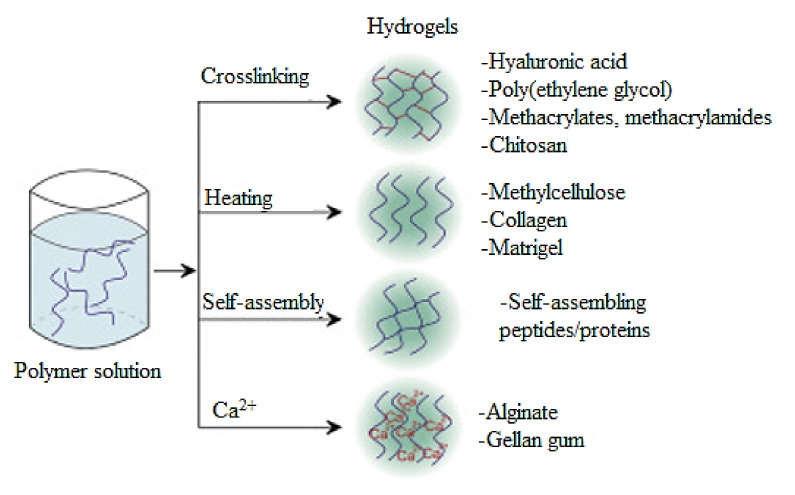
The main crosslinking methods are used for the development of hydrogels [78].

**Figure 3:**
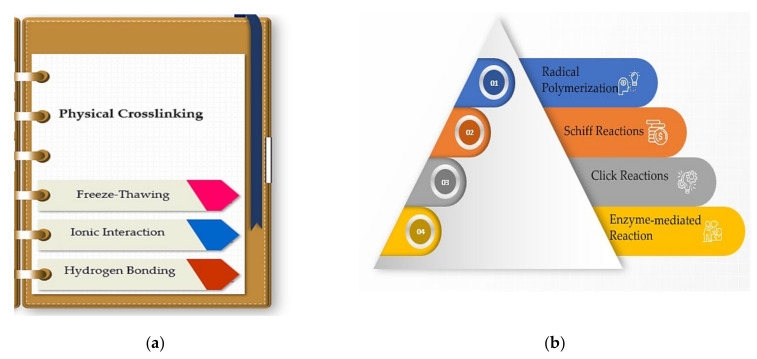
The main methods of (**a**) physical and (**b**) chemical crosslinking of hydrogels.

The hydrogels are defined as hydrated systems where the hydrophilic units are crosslinked, and therefore provide a compact structure known as a polymeric network. The gelation process occurs through a chemical or physical crosslinking [76,77]. In this regard, Figure 2 and Figure 3 present the main crosslinking methods used to develop hydrogels.

Physical crosslinking represents one of the most used crosslinking methods for developing polymeric scaffolds. The resulting materials obtained from physical crosslinking are physical or reversible hydrogels. They can also be dissolved in the selected environment; the degree of dissolution can be changed by varying the pH or temperature [79]. Nevertheless, the developed polymeric systems provide poor mechanical features and are structurally unstable [80].

### 4.1. Physical Crosslinking Methods

#### 4.1.1. Freeze-Thawing Method

This technique is one of the most used physical crosslinking methods that can be achieved by using repeated freeze-thaw cycles. This mechanism implies microcrystals formation in the polymeric structure due to the polymer’s repeated freezing and thawing cycles. The process diminishes the polymer chain spaces and increases the polymer concentration. In this regard, Sharma et al. present the possibility of developing hydrogels based on PVA using the freeze-thaw method. These are presented in the form of interconnected networks, which possess superior properties in terms of porosity and elasticity, compared to their obtaining by other methods [80,81,82,83].

#### 4.1.2. Ionic Interactions

In this category are considered crosslinked ionic polymers formed by the addition of different di-tri valents. This method is characterized by the gelling of a polyelectrolyte solution that contains multivalent ions with opposite charges. Examples of hydrogels belonging to this category are poly-[di(carboxylatophenoxy) phosphazene] calcium salt and chitosan/glycerol phosphate salt [84].

#### 4.1.3. Hydrogen Bonding

Hydrogen bonds are significant physical interactions that lead to obtaining tertiary and secondary structures. Determined by the degree of protonation and the chemical environment that allows polar functional groups’ protonation, this intermolecular interaction can confer soft materials with self-healing properties. A good example of these interactions is the development of hybrid hydrogels by Zhang et al. These researchers developed hydrogels based on gelatin methacrylate containing tannic acid, which was used to demonstrate its excellent bonding capacity [85]. The main disadvantages of these physical crosslinking methods are the lack of adequate strength and mechanical stability. To overcome this disadvantage, Parhi et al. focused mainly on chemical crosslinking. Thus, the chemically obtained hydrogels are also known as permanent hydrogels, obtained through the addition of chemical agents that bind polymers through covalent interactions. Moreover, the swelling rate of the obtained hydrogel depends mainly on the concentration of the crosslinking agent. Considering these aspects, Majid et al. developed a polymeric hydrogel based on acrylic acid and gelatin constructed by free radical polymerization. The hydrogel was crosslinked using ethylene glycol dimethacrylate, and ammonium persulfate as initiator. After thorough investigations, it was concluded that the polymeric system’s swelling capacity depends on the concentration of the chosen monomer, the crosslinking agent, and the used polymer [30,81]. Therefore, covalent crosslinking hydrogels are characterized as networks with high mechanical strength because the covalent bonds are irreversible [82,83,84].

### 4.2. Chemical Crosslinking Methods

#### 4.2.1. Radical Polymerization

Free radical polymerization is generally used to obtain covalently crosslinked hydrogels, by using vinyl monomers or their functionalized version, with the addition of radical initiator and crosslinker. The main benefit of this approach is represented by the availability of numerous monomers. In this regard, Mohamed et al. describe the possibility to apply natural methacrylate or acrylate polymers, such as methacrylate hyaluronic acid, methacrylate gelatin, or methacrylate heparin, to obtain a hydrogel with outstanding bioactivity. However, the produced materials require a photo or thermal stimuli to initiate crosslinking that may induce cytotoxicity [45].

#### 4.2.2. Schiff Base Reaction

The formation of hydrogels through Schiff base reaction implies a reaction between amino and aldehyde groups. These are functional groups of the polymers that are involved in the crosslinking process, under light conditions. Natural polymers containing aldehyde can be produced by partial oxidation of polysaccharides (e.g., dextran, hyaluronic acid, gum arabic, chondroitin sulfate, and many others), and their subsequent reactions with natural or synthetic polymers containing amino groups. This sequence of reactions induces hydrogel formation. Similarly, the reaction of polymers containing ketones or aldehydes with a polymer that includes hydroxylamine can produce hydrogels by forming oxime bonds. The reaction takes place at a high rate, without the use of a catalyst. Another example is represented by the reaction between amino or hydroxyl groups of the polymers and isothiocyanate, for the preparation of the hydrogels [45].

#### 4.2.3. Click Reactions

Click reactions are extremely effective reactions that allow hydrogel formation with a well-defined structure in physiological or mild conditions. For instance, click reactions include Diels-Alder reaction, alkyne-azide cycloaddition, thiolene reaction, and many others. Alkyne-azide cycloaddition of catalyzed copper is frequently used in bioconjugation, but copper cytotoxicity averts the use in regenerative medicine. Another class of click reactions is represented by Diels-Alder reactions which occur between dienophiles and dienes for substituted cyclohexene formation. They are commonly applied for hydrogel development in aqueous solutions without using catalysts. Though, the slow reaction’s kinetics prevent their use in processes that request the quick formation of hydrogels (e.g., in situ implantation) [45].

#### 4.2.4. Enzymatic Crosslinking

Another strategy used for crosslinking is represented by an enzymatic reaction. Polymers that contain enzymatically reactive regions such as tyrosine, tyramine, aminophenol, and dopamine undergo fast in situ gelation during oxidation by using hydrogen peroxide in the presence of catalysts such as horseradish peroxidase, as reported by Moeini et al. [45]. Additionally, chemical crosslinking is well known as a common pathway to reduce the degradation rate of the scaffold. The most used agents to stabilize the polymers are glutaraldehyde (GA) and formaldehyde (FA). Their major disadvantage is represented by the possible appearance of potential inflammatory response and cytotoxicity. To overcome these major drawbacks and improve the stability of the polymers, natural and non-cytotoxic crosslinkers such as curcumin, genipin (GE), tannic acid (TA), and N-(3-Dimethylaminopropyl)-N′-ethylcarbodiimide hydrochloride (EDC) could be selected [85,86,87].

### 4.3. The Influence of Crosslinking Agents

Even though the crosslinking process is performed to enhance hydrogels’ properties, the used synthetic crosslinking agents could produce undesirable modifications of the polymeric structure or even cause increased cytotoxicity [88]. Many studies confirmed that the use of GA caused increased cytotoxicity for both preclinical and clinical studies. This effect could be determined by the functional groups that cannot participate in the reaction or that are produced during enzymatic degradation. For example, Gough et al. performed the GA cytotoxicity assessment, used to crosslink collagen biomaterials. It was concluded that the presence of GA led to considerate apoptosis. Hence, the use of natural crosslinkers could surpass the toxicity issue [89]. In this regard, Jiang et al. have reported that the use of citric acid as a crosslinker can enhance the stability and mechanical properties of scaffolds, without compromising the cytocompatibility of the biomaterial. Further, citric acid allowed ester bond formation and improved the functionality of the material to provide increased availability for bioconjugation and enhanced hemocompatibility [90]. GE (a natural aglycone derived from geniposide) could act as another suitable example for natural crosslinking agents being known for its versatile cross-linking properties due to amino groups found in various proteins, and extremely low cellular toxicity [91]. Moreover, Sung et al. succeeded to demonstrate that GE, in comparison with GA, could provide a reduced cytotoxic effect [92].

Another good example of a natural crosslinking agent is represented by TA, the most important constituent of hydrolysable tannins. This compound has been intensively studied for its beneficial effects, such as good antimicrobial, antioxidant, anti-inflammatory, and antiviral properties [89]. Brazdaru et al. concluded that TA possessed the highest affinity as a crosslinker for collagen biomaterials without causing toxicity [93]. Additionally, natural products such as curcumin have the capacity to couple the functional groups located in the polymer chain. Hanafy et al. demonstrated that curcumin encapsulation could aid the production of bio crosslinking agents for mucoadhesive polymers. In this case, it was concluded that curcumin could act as a suitable natural crosslinker for the optimization of hydrogel-based scaffolds, and at the same time, inhibit the proliferation of cancerous cells [94,95].

Similarly, Reddy et al. concluded that proanthocyanidin (PA), a natural compound found in grape seeds, can be used as a crosslinker. Studies have shown that PA increased the resistance to enzymatic degradation and thermal resistance of collagen films, without interfering in the cytocompatibility of the material. Further, after several weeks of implantation, it was concluded that crosslinked films presented a significant penetration of fibroblasts, without any cytotoxic effect [88]

## 5. Properties of Hydrogel-Based Scaffolds

According to numerous studies, it was proved that scaffolds must meet several characteristics to be used for tissue engineering. Thus, these polymeric networks must have characteristics similar to ones of the regenerated tissue. In the following section, we will describe the most essential properties for hydrogels applied in tissue engineering.

### 5.1. Porosity

One of the most important properties of polymeric scaffolds is porosity. This property is essential for biomaterial development, allowing the flow of oxygen and nutrients into cells, and the removal of the entire cell debris. Additionally, cell migration and tissue integration are influenced by the porous structure of the scaffold. Further, the size of the pores is crucial for cells penetration depth within the scaffold.

**Figure 4:**
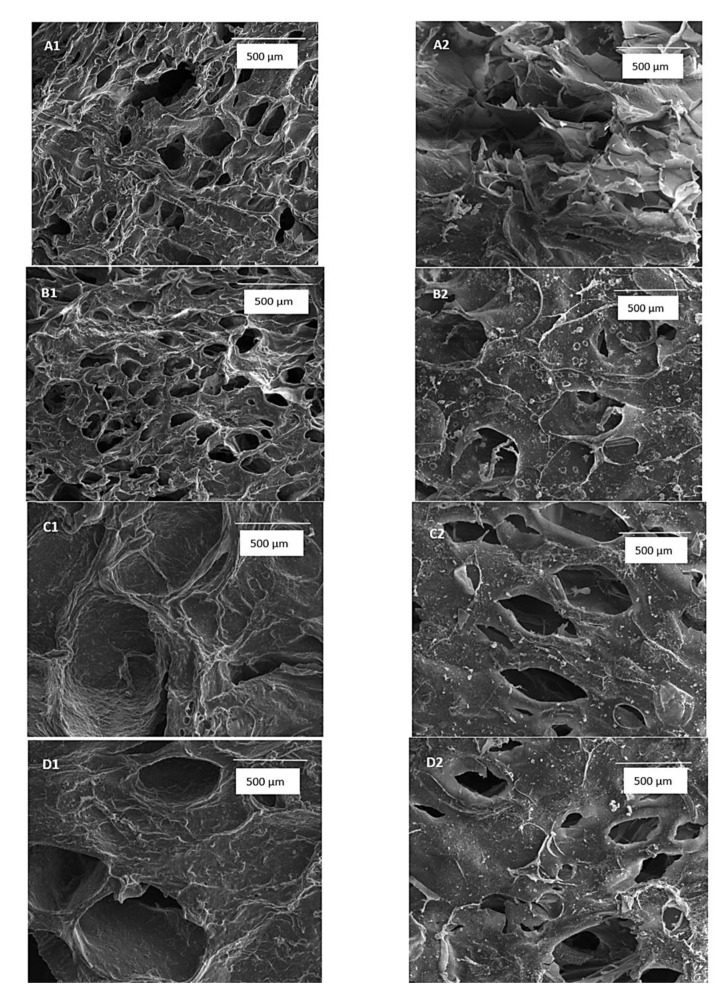
Scanning electron microscopy of hydrogels (**A**) without TA; (**B**) 10% TA; (**C**) 20% TA and (**D**) 30% TA, (1) before immersion in SBF and (2) after 7 days at 150× magnification [97].

Many studies concluded that pores with sizes between 20–125 μm in the dermal collagen-glycosaminoglycan matrices were the key to controlling contraction inhibition. The microscale characteristics of the individual pores and the groups formed between them are important for the control of the microarchitecture, cell aggregation, orientation, and function. The pores <5 μm are essential for neovascularization, while the ones between 5–15 μm influence the ingrowth of fibroblasts. Moreover, the pores with sizes between 20–125 μm encourage the infiltration of the adult mammalian cell. Additionally, the pores between 40–100 μm facilitate osteoid ingrowth and the ones with sizes >500 μm are necessary for fibrovascular tissue growth. Hence, the precise control of scaffolds’ porosity can influence the biomaterial-tissue interaction [96]. In this regard, Kaczmarek et al. demonstrated that the combination of sodium alginate and TA provided a porous structure with larger interconnected pores where the crosslinking agent improves the material stability and permitted biomineralization, as shown in Figure 4 [97]. With the immersion of the hydrogels in simulated body fluid (SBF), a biomineralization process has been observed on their surface. By performing bioerosion studies, it was demonstrated that the addition of TA improved their stability. Also, it was concluded that the porosity increased with the addition of the active compound. In addition, due to the high TA concentration, the antibacterial activity of the polymeric scaffold is increased.

**Figure 5:**
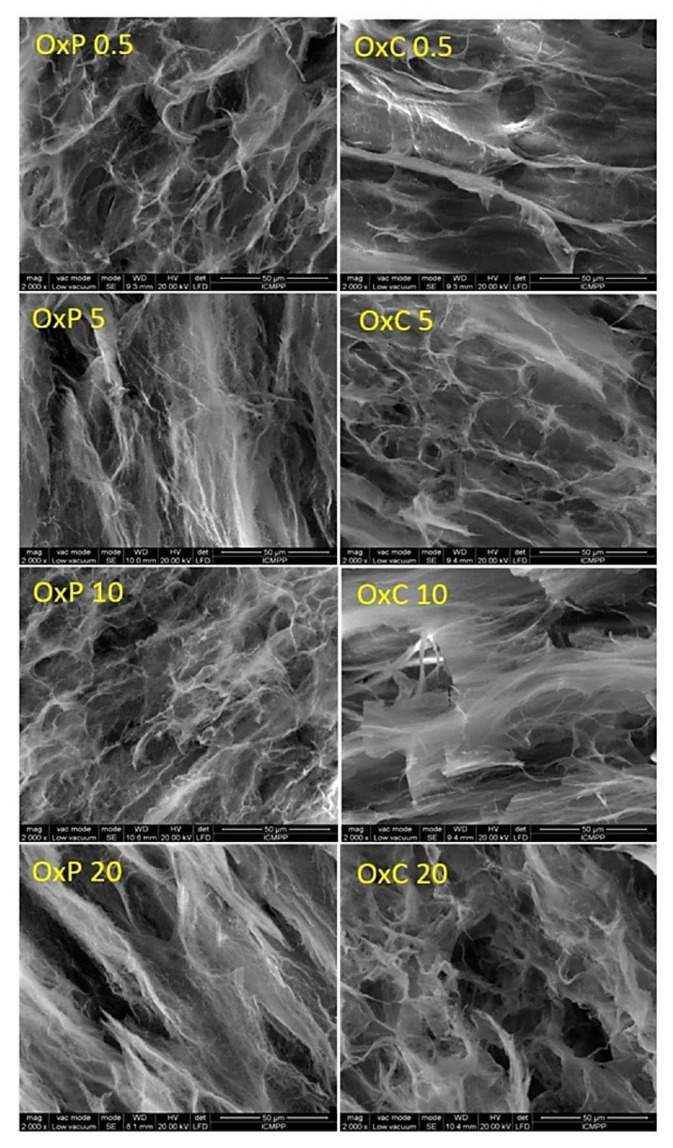
SEM images (2000× magnification) of PVA scaffolds with oxidized pullulan (0.5% (OxP 0.5), 5% (OxP 5), 10% (OxP 10) and 20% (OxP 20)) and tricarboxy cellulose (0.5% (OxC 0.5), 5% (OxC 5), 10% (OxC 10) and 20% (OxC 20)) respectively [98].

Another representative study about hydrogels morphology is explained by the scaffold developed by Baron et al. based on PVA and oxidized cellulose (OxC)/oxidized pullulan (OxP). The composite hydrogels with different oxidized polysaccharides content have been obtained using the freezing/thawing method. In this study, the hydrogels showed the homogeneous distribution of both OxP and OxC into the PVA matrix, confirming an excellent distribution of the oxidized polysaccharides. As shown in all SEM micrographs from Figure 5, the hydrogels possess an increased porosity, with large, interconnected pores ranging between 14–46 μm, proving the successful incorporation of OxP and OxC.

### 5.2. Mechanical Strength and Stiffness

Moreover, polymeric scaffolds should have a mechanical strength similar to the natural tissue to regenerate or provide support [99]. In this direction, the mechanical properties must correspond to a specific tissue, to enhance cell adhesion and accurately fill in the damaged tissue. Further, it is well known that the rigidity of the matrix influences stem cells differentiation. Following the mechanical properties, suitable biochemical properties influence the specific protein sequences present in the ECM and enhance the interaction between cells and scaffolds. Each type of tissue accomplishes different functions and therefore, it is characterized by different morphologies, stiffness, physiology, and biochemistry. Considering the previously mentioned aspects, the necessary mechanical requirements for each biomaterial are specific to the targeted type of tissue [100].

From the macroscopic point of review, the mechanical properties are necessary to maintain the stability of the scaffold to bear loading and substitute the defects. At the microscopic scale, the mechanical signals impact cell activity and function. Thus, Li et al. concluded that matrix stiffness can affect cell proliferation, differentiation, and migration. The stiffness of the polymeric materials can be controlled by changing crosslinking length, density, and molecular weight of the precursors [101]. Further, the exposure of cells to more rigid substrates leads to an increased elastic modulus, especially in their plasma membrane, with a much better-organized actin cytoskeleton. Moreover, Vedadghavami et al. demonstrated that cells grown on stiffer structures proliferate quicker and migrate more slowly than cells on softer substrates [102].

### 5.3. Swelling Behavior

Pinelli et al. reported that the most important characteristic of hydrogels is their capacity to swell when the system is placed in contact with water or a thermodynamically compatible solvent. When the hydrogel structure encounters the molecules of the solvent, it enters the polymeric network. Due to hydration, the polymeric chains relax, and, as a result, the entire system expands. This process is sustained by the present osmotic forces, while the elastic force of the hydrogel balances the polymer network and hinders its deformation leading to equilibrium (equilibrium water content) without additional swelling. Further, swelling capacity is also affected by many components of the hydrogel network, such as the functional groups present in the polymeric chains, the degree of crosslinking, the polymer nature, and many others. All these elements are vital parameters that change the swelling capacity of the hydrogel [103]. Therefore, it was concluded that if the hydrogels are more rigid, the swelling capacity of the material is lower. Consequently, Lan et al. reported that hydrogels’ porosity and inner pore size can directly influence the swelling rate, exchange capacity, and mechanical properties [104].

### 5.4. Adhesion

The adhesion between hydrogels and tissue substrates is another important feature. This property is described as the formation of junctions and connections between two surfaces. Hydrogel-based scaffolds are known as inherently hydrated materials and their adhesion relies on sparse and loose adhesion junctions, which are surrounded by water. The bond formation at hydrogel’s surface can be complex as partially dissociated and polar water molecules can shield functional groups through Coulomb and van der Waals interactions. The adherent polymeric chains interact with free water molecules and therefore exchange molecular neighbors instead of forming stable connections with the other adherent surface. Furthermore, adhesion can occur when adhesion junctions are formed at the interface of two or more adherents. Their stability, reversibility, mechanical strength, and response to environmental stimuli are strictly related to the adhesion junction’s design. The junctions can be formed through permanent, dynamic covalent, or noncovalent bond formation. The most important characteristic of this property is that the junction chemistries must be compatible and nontoxic with the biological processes, and therefore do not affect cell or tissue functions. If direct interactions with the human body are involved, the polymeric scaffold should not trigger any immune response or inflammation. Further, the designed hydrogel should be able to deform, in accordance with the tissue of interest and, if possible, to match the mechanical properties of the tissue.

Therefore, it can be concluded that adhesion generally occurs through the formation of physical or chemical interactions between the hydrogel-tissue surface. The ability to form the abovementioned junctions remains challenging as the interfaces are wet and deformable environments [105].

### 5.5. Biodegradability

Biodegradability represents another essential property for hydrogels’ development as they require controlled in vivo degradation and resorption. This feature depends mainly on mass dissolution, based on several processes, such as photolysis, hydrolysis, separation, or even a combination of these techniques [106]. The degradation of the scaffolds is known as a chemical process, but it can be determined also by a dynamically physical stimulus that affects cell function and behavior The main purpose of this process is to provide the ability to mimic ECM and enhance tissue regeneration. However, scaffold degradation is commonly accompanied by a decrease in stiffness, which makes it difficult to discriminate the influences of degradation and stiffness. To decouple the influence of the mechanical properties of biodegradable hydrogels, the hydrogels are designed to degrade while their mechanical properties remain unchanged [101]. In this direction, Unagolla et al. confirmed that material degradation creates more space that allows proliferation, migration of cells, and blood vessels infiltration. The spatio-temporal development of this type of biomaterials still presents a challenge at the moment [106].

### 5.6. Biocompatibility

The purpose of assessing any hydrogel’s biocompatibility is to limit the toxic effects induced in the organism. Therefore, the evaluation of scaffolds for assessing biological responses that may cause unwanted damage or side effects to the host is particularly important. The three major factors that should be considered are the healing process, inflammation, and the immune response known as immunotoxicity. For example, Naahidi et al. related that hydrogels are preferred in this field due to their natural compositional and structural similarities with the ECM. Therefore, hydrogels can be designed to control molecular responses, cell attachment, structural integrity, biocompatibility, and biodegradability [107]. Also, for scaffolds that are conceptualized for tissue regeneration, it is necessary to avoid the use of materials that could cause immune responses and inflammation. Another important feature in the conceptualization of a polymeric scaffold is cellular affinity.

**Figure 6:**
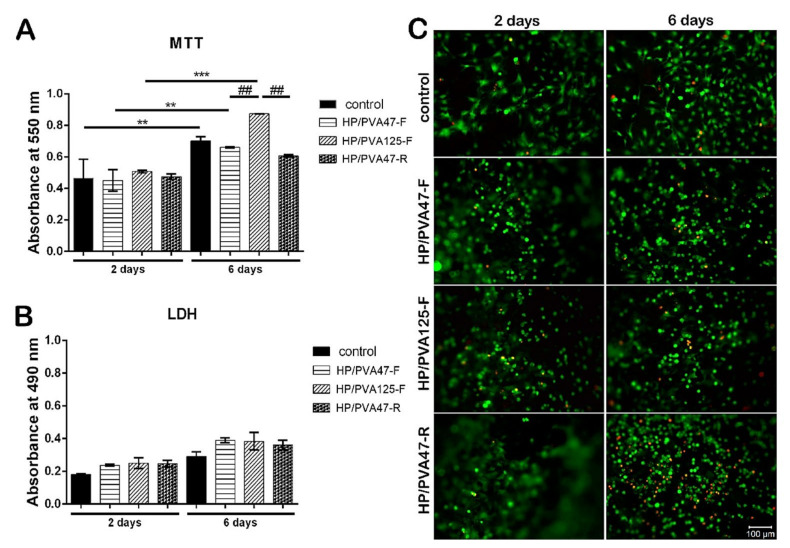
Cell viability and proliferation assessment: (**A**) using MTT assay (** *p* > 0.01 and *** *p* > 0.001); (**B**) LDH assay for cytotoxicity evaluation; (**C**) Confocal microscopy: dead (red) and live (green) cells, scale bar 100 µm using L929 murine fibroblast cells [111].

Because cell participation is essential in tissue regeneration, Mellati et al. demonstrated that the developed material should easily facilitate attachment or incorporation [108,109,110]. Another good example of this feature is the material developed by Samoila et al. as shown in Figure 6 [111].

As mentioned before, Figure 6 presents the biocompatibility assessment results of hydrogels based on PVA (with different molecular weight (Mw): 47,000 g/mol and 125,000 g/mol) and pullulan (HP) using two crosslinking methods (chemical method using trisodium trimetaphosphate (STMP)-at room temperature (R) and freeze-thawing method (F)). As shown in Figure 6A, all samples exhibited increased biocompatibility. On day 6, the hydrogels exhibited different proliferation levels depending on the crosslinking route or molecular weight. The control presented an increased proliferation rate (** *p* < 0.01) after 6 days, compared with day 2. Further, for the samples that were synthesized using freeze-thawing cycles, it was concluded that they provided increased viability at first, and at 6 days displayed a statistically significant elevation of proliferation compared with the chemically crosslinked materials. The samples which were obtained using the highest PVA Mw showed the highest proliferation rate from all the developed samples. Figure 6B presents the cytotoxicity assessment of composite materials through lactate dehydrogenase (LDH) assay. In this respect, the samples presented a similar behavior as the control. After 6 days, there is only a small cytotoxicity increase. Also, it was concluded that the developed samples did not exhibit any significant cytotoxic effect. Figure 6C presents the viability assessment using Live/Dead staining where the live cells are significantly higher than for the dead cells for all samples. Moreover, the cells presented a uniform distribution, and after 6 days, it was concluded that the sample with the highest Mw provided the highest viability. Further, chemical crosslinking could negatively influence the viability of fibroblastic cells as, after 6 days, the number of dead cells increased.

Another particularly important property of tissues regeneration is represented by the seeding of exogenous cells into developed scaffolds and the generation of an environment favorable for cell migration to the damaged region. The capacity of hydrogels to be integrated into tissues is one of the main qualities that recommend them in tissue engineering. In this direction, adhesion between hydrogels and tissue is vital to prevent treatment failure, caused by the detachment of both components. Considering these aspects, Sani et al. explained that this adhesion is important to promote material integration in tissues during regeneration [112,113]. Feasibility is also particularly important because injectable hydrogels provide great advantages in practical applications [114]. In the last few decades, hydrogels’ development has gained great interest due to their ability to encapsulate cells and bioactive molecules, but also due to the efficient mass transfer achieved through diffusion [115]. Polymeric chains can penetrate and join the tissue to obtain a semi-permanent and adhesive bond. The interaction is based on the polymer concentration, molecular weight, and the similarity between tissue and polymers. Thus, the adhesive force is improved to reach saturation due to the polymer’s increased molecular weight. The same principle is applied to increase the concentration of polymers used. However, Thi et al. concluded that if the polymeric solution is highly concentrated, the concentration can interfere with the flexibility of the polymeric chains and therefore reduce diffusion, thus decreasing their adhesion property. The adhesion characteristic of hydrogels and tissues should be similar in terms of molecular structure, branched chains, and their hydrophilicity, to obtain a facile diffusion [116].

## 6. New Directions of Hydrogels for Biomedical Applications

In recent years, numerous studies have shown that hydrogel-based scaffolds are excellent substrates that can be applied for cell differentiation and transplantation, controlled drug delivery, endogenous regeneration, wound healing, and bioprosthetic [117]. Hence, the main applications of hydrogels can be seen in Table 2.

Each hydrogel must fulfill specific requirements to be applied in biomedical fields. As an example, regeneration of the skin and cartilage requires control over mechanical features (strength, stiffness, elongation) and malleability in complex shapes. Controlled release systems of active ingredients aim to maintain the bioactive agent inside the system until it reaches the site, where it triggers a controlled release [128,129].

### 6.1. Skin Tissue Engineering

Skin regeneration can be accomplished using substituents such as dressings containing allogeneic skin cells, grafts, or even cellular scaffolds used for durable applications [130,131]. Hydrogels have received special attention as biomaterials for wound management and skin regeneration that can also be used in tissue engineering [132,133,134]. In this regard, films based on nanocomposite hydrogels can promote wound healing and protect damaged tissue from external factors [135]. The humid environment created by hydrogels improves the wound healing process. It allows an efficient debridement (the removal of foreign material and necrotic tissue) due to the inner absorption capacity. Hence, Koehler et al. related that hydrogel-permeable structures allow water, O_2_, and CO_2_ exchange, permitting the tissue to “breathe” [136]. Xiang et al. concluded that dressings based on hydrogel create a moist environment, simplifying the healing steps. The limited adhesion of these polymeric materials and the moisturized bed of the wound facilitates the dressing’s removal without any secondary injuries. Also, hydrogels should be able to significantly diminish the risk of infection and discomfort caused by dressing replacement. Certain dressings are constructed to be transparent and therefore facilitate the clinical evaluation of wound healing without removing the dressing, avoiding any additional injury [137].

For example, Mohandas et al. obtained composite bandages by preparing hydrogels based on sodium alginate/chitosan loaded with different concentrations of zinc oxide nanoparticles (ZnO NPs). The hydrogels exhibited significant biocompatibility, hemostatic ability, and antimicrobial activity against different strains, namely *Staphylococcus aureus*, and *Escherichia coli*. Furthermore, Raguvaran et al. incorporated ZnO NPs in acacia gum/sodium alginate hydrogels, which provided an enhanced antibacterial activity against *Pseudomonas aerigunosa* and *Bacillus cereus* [138]. Moreover, Martins et al. synthesized hydrogels based on alginate/N, N, N-trimethyl chitosan. The developed specimens were loaded with gold nanoparticles (Au NPs) and presented increased antimicrobial activity and biocompatibility, making them potential wound dressings. To improve antimicrobial activity and reduce scarring, Sadiva et al. added tetracycline hydrochloride in chitosan/PEG/polyvinyl pyrrolidone hydrogels. The composite hydrogel exhibited superior antimicrobial properties on different bacterial strains. Tetracycline provided a proper barrier against bacterial infection, and chitosan was able to promote wound healing with minimal scarring [139].

**Figure 7:**
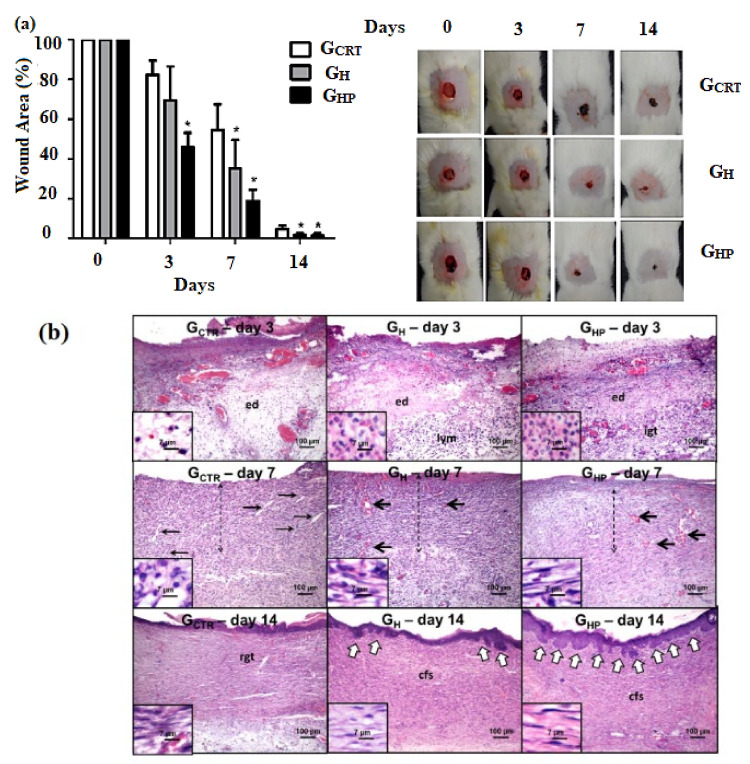
(**a**) Wound surface percentage for: Control Group (G_CTR_), group with hydrogel sodium alginate/gelatin (80:20) (G_H_), and group hydrogel with AgNP 4 mM AgNO_3_ (G_HP_) with mean ± S.E. * *p* < 0.05 in relation to G_HP_, G_CTR_ and G_H_, respectively (*n* = 21/group). (**b**) Photomicrographs of histological sections stained with hematoxylin/eosin (scale = 100 µm) [140].

Resende Diniz et al. demonstrated the in vitro evaluation of hydrogels based on gelatin/sodium alginate containing Ag NPs for wound healing evaluation, as shown in Figure 7. The samples were divided into control (G_CTR_), hydrogel based on sodium alginate/gelatin (80:20) (G_H_), and the hydrogels with AgNPs (G_HP_) [140]. From Figure 7a, it can also be concluded that the wound size is slowly decreasing in time, where both groups that contain hydrogels have a significant decrease regarding the wound area compared with the control group (*p < 0.05). The addition of NPs is mostly highlighted on day 3 where the wound size is reduced to ~40% and for the control group ~17%. During day 7, it could also be observed that the most successful materials are represented by the group which contains nanoparticles-G_HP_ as the size of the wound is decreased by ~80%, followed by the group treated with only hydrogels G_H_ with a decrease of ~60%.

Further, after 14 days, granulation tissue formation could be associated with the effect of hydrogels with and without Ag NPs. The addition of Ag NPs could enhance the antimicrobial activity, and the wound area would be reduced much faster than the control group. Observing the results at day 3 in Figure 7b, the micrograph displays dense edema (ed) and polymorphonuclear neutrophils infiltration, where immature granulation tissue (IGT) and lymphocyte-rich infiltrate (LYM) can both be detected for G_HP_ and G_H_ groups. For all groups, macrophages can be observed throughout the wound. Additionally, lymphocytes (dark round nuclei) and polymorphonuclear neutrophils (small lobular nuclei) are highlighted in higher magnification (respectively in the small sections from Figure 7b). After day 7, the dashed arrows indicate granulation tissue formation with different degrees of maturation. The stromal cells are dispersed through the wound in a parallel arrangement, and for G_HP_’s maturation process is more advanced than for the other groups. The thin arrows highlight slit-shaped and irregular capillary blood vessels observed at the edges of G_CTR_. On the other hand, the thick arrows highlight dilated hyperemic vessels in both G_HP_ and G_H_ groups, where the concentration of inflammatory cells is reduced for the G_HP_ group. Moreover, after 14 days, cellular primary fibrous scars (cfs) can be observed in both G_HP_ and G_H_ groups, while the control group contains residual granulation tissue.

Another clinical study accomplished by Mirzaei et al., it was evaluated the antimicrobial activity of alginate hydrogel that contained honey as an active principle. In this regard, the in vivo antimicrobial activity of honey has been firstly assessed by using Wistar rats (female, 6–8 weeks) against *A. baumannii, Klebsiella pneumoniae, P. aeruginosa,* or *S. aureus* strains. After 24 h, post-burn induction, the rats received 5 g of hydrogel twice per day. Hence, it was concluded that alginate hydrogel shortened the closure period of infected wounds with all pathogens tested, compared to untreated models [141]. A similar study has been reported by Bagher et al. where they demonstrated the successful application of alginate/chitosan hydrogels containing hesperidin in wound healing. The clinical evaluation has been accomplished using rat models (2 weeks). The obtained hydrogels possessed a 91.2% porosity, with interconnected pores, suitable swelling ability, and proper biodegradability, confirmed by over 80% weight loss after 2 weeks. The wound healing evaluation proved that hydrogels formulation provided a quicker healing period, compared with commercial gauze [142]. Similar findings have also been reported by Ehterami et al. where the alginate/chitosan hydrogels have been loaded with Alpha-tocopherol. The developed scaffolds provided an 89.2% porosity, with interconnected pores and similar biodegradability. In this case, the same effect related to wound closure has been observed, and for the group, treated with hydrogels, granulation tissue, and neo-tissue could be clearly observed [143]. Further clinical studies of polymeric scaffolds applied for wound healing applications which were evaluated on different subjects are presented in Table 3.

### 6.2. Bone Tissue Engineering

**Figure 8:**
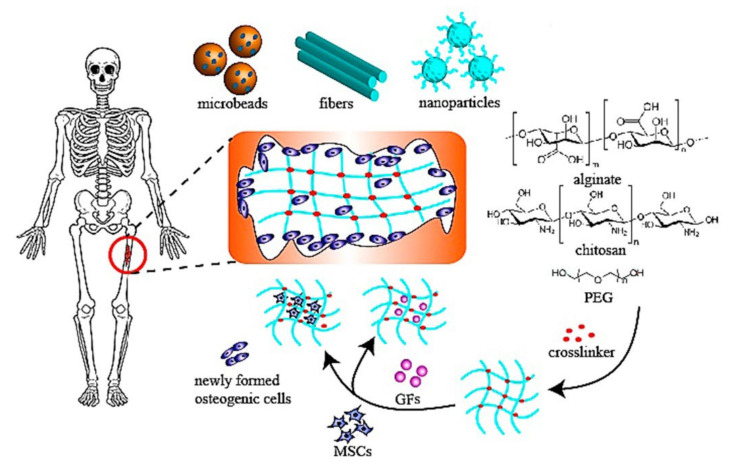
The main applications that can be applied in bone regeneration [158].

To promote enhanced bone regeneration, hydrogels can be considered a potential solution for cell release and the administration of active ingredients. They provide a hydrophilic environment that supports bone growth and cell proliferation. In addition, hydrogels can be designed to obtain the anticipated geometry for injection procedure or implantation. The porosity, degradation rate, or release profile could be regulated by adjusting the crosslinking degree or even synthesis method. Figure 8 shows the main applications of hydrogels used in tissue engineering for the treatment of bone defects [158]. In this regard, Bai et al. highlighted that hydrogels applied in bone regeneration must fulfill the following characteristics: osteoconductivity, osteoinductivity, osteogenic capacity, osteocompatibility, non-immunogenicity, and non-cytotoxicity to avoid inflammatory response. Moreover, they can mimic ECM, facilitate cell propagation, adhesion, and, finally, osteogenic differentiation at the implantation site. Another important feature of hydrogels is the ability to be degradable, synchronizing with the bone growth, making enough space for bone formation, structural stability, and tunable high mechanical strength. Hydrogels must also have suitable pore sizes, where the interconnected porosity could be improved by changing the concentration, degree of crosslinkers, and the type of polymers. These processes are optimized to control the release rate of bioactive molecules, improve cellular interactions, and allow the exchange of oxygen, nutrients, and metabolic wastes into hydrogels [158].

**Figure 9:**
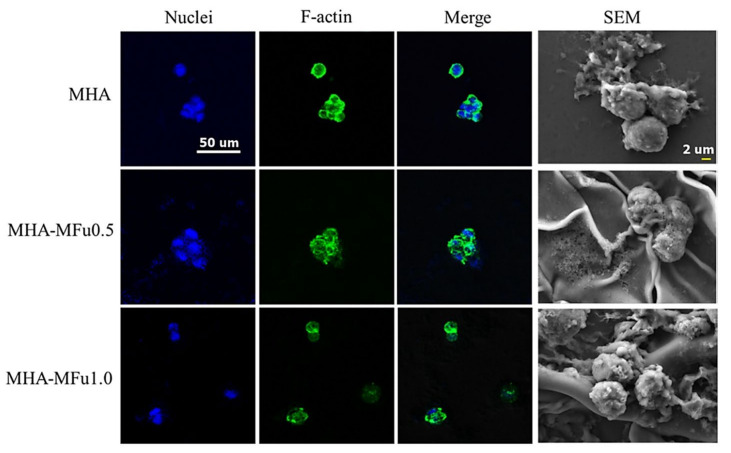
SEM and immunofluorescence assessment of cell morphology on MHA-MFu and MHA hydrogels after 7 days. Scale bar: 2 μm for SEM and 50 μm for immunofluorescence images [159].

As shown in Figure 9, Hsu et al. combined hyaluronan, and fucoidan (polysaccharide rich in sulfate) grafted using methacrylic groups.

The concentration of methacrylate-fucoidan (MFu) was variated (0, 0.5, and 1%). The obtained hydrogels were assessed from a cell adhesion and proliferation point of view. In this regard, MG63 osteoblasts were stained with F-actin (green), and cell nuclei were stained with DAPI (blue). It was concluded that the cells attached on hydrogels that contained 0.5 and 1% MFu exhibited spherical morphologies, like the ones attached on the hydrogel which didn’t contain MFu. After proper evaluation, it was demonstrated that the addition of fucoidan did not affect cell morphology. By analyzing the SEM images, it was also concluded that MG63 cells successfully adhered to the MHA (methacrylate-hyaluronan) polymeric scaffold. Furthermore, the rough and uneven surface of the hydrogels which contained MFu permitted an increased attachment for MG63 cells and could lead to higher ECM production [159].

Another good example of this kind of polymeric scaffolds is represented by the hydrogels obtained by Heo et al. They introduced Au NPs in hydrogels based on gelatin. The hydrogels were further reinforced with fibrous scaffolds based on polylactide. Regarding the compressive modulus, the scaffolds obtained similar values with human mandibular bone. Additionally, the hybrid structures enhanced the gene expression of osteogenic factors. Further, the scaffold developed by Thompson et al. could be an excellent alternative to replace allografts. In this case, chondrogenically primed mesenchymal stem cells were embedded in scaffolds and promoted an improved repair of bone defects with critical sizes [8]. Furthermore, it is well known that in hydrogels, the biomechanical properties and adhesion sites can be modified within the gel to improve therapeutic efficacy and cell viability. Vegas et al. developed a hydrogel-based on chemically modified alginate known as triazole-thiomorphiline dioxide. The obtained material was used as implant coating to successfully transplant hESC-derived β cells into diabetic patients mice, which were immune to streptozotocin [160]. Chen et al. developed a temperature-sensitive injectable hydrogel based on poly (D, L-lactic-co-glycolic acid) and b-methoxy poly (ethylene glycol) to incorporate the vascular endothelial growth factor (VEGF), needed to form the process of neovascularization. The hydrogel presence causes a delay in growth factors to release in contrast to the microspheres, while the in vivo results recommended the feasibility of their application for bone regeneration and vascularization of femoral head necrosis [161].

### 6.3. Cartilaginous Tissue Engineering

Regeneration and repair of cartilaginous defects are also one of the most important tissue engineering applications. Hydrogel-based scaffolds can be widely used for biomedical applications due to their tunable properties. The mechanism by which the structure of articular cartilage is formed is a key factor for regeneration. Both natural and synthetic polymers can be used to develop these types of scaffolds. These polymers play a crucial role in cell attachment, migration, and differentiation [162]. For example, Ahmed et al. described that chitosan improves chondrogenic differentiation into bone marrow mesenchymal stem cells through metal proteins inactivation and interrupts collagen degradation. The obtained scaffold was modified with biologically active hydroxyapatite (HA), thus increasing the biocompatibility and biodegradability of the material to aid cartilage repair and formation. Further, Visser et al. obtained gelatin/hyaluronic acid hydrogels reinforced with polycaprolactone scaffolds. The rigidity of the developed structure approached the values of articular cartilage while sustaining physiologically appropriate elasticity [8].

Another study reported the use of type II collagen in cartilage formation. In this direction, polycaprolactone and chitosan-based scaffolds loaded with collagen have been developed and successfully applied in cartilage tissue engineering [163]. Cavalu et al. described the development of composites based on PVA, reinforced with HA and Se-doped TiO_2_ NPs using the freeze-thawing method [164]. The samples were coded based on the calcination time of TiO_2_ (400, 600, and 800 °C for 2 h). The obtained scaffolds provide a honeycomb structure containing both Se-doped TiO_2_ and HA NPs. It was concluded that HA presence affects the morphology of the hydrogel to a porous and fibrous matrix, giving important advantages such as interconnected porosity, suitable to improve vascularization. MTT assay (3-[4,5-dimethylthiazol-2-yl]-2,5-diphenyl-tetrazolium bromide) has been performed to assess proliferation rate and cell viability.

**Figure 10:**
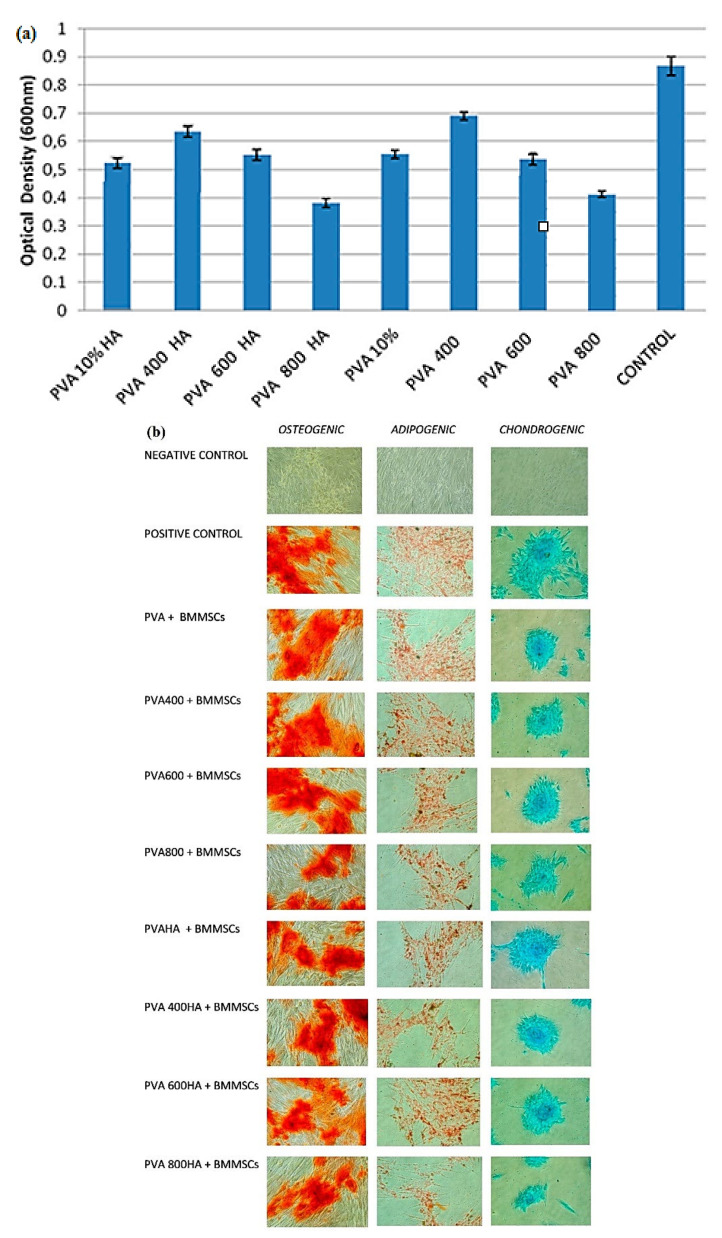
Hydrogels based on PVA/Se-doped TiO_2_ NPs/HA [164] (**a**) MTT assay of BMMSCs with PVA-based composites after 72 h incubation, and (**b**) Differentiation potential of BMMSCs to osteogenic, adipogenic, and chondrogenic lineages, of PVA nanocomposites after 72 h of incubation.

Examining Figure 10a, it has been concluded that all samples provided good cellular viability compared with the control sample. Additionally, except for the composites which contained nanoparticles calcinated at 800 °C, all composites exhibited improved viability. Figure 10b presents the differentiation potential of bone marrow mesenchymal stem cells (BMMSCs) to chondrogenic, adipogenic, and osteogenic lineages using the developed composites. Hence, it was concluded that the hydrogels obtained an improved chondrogenic and osteogenic differentiation compared to adipogenic differentiation. The fibroblast-like morphologies of BMMSCs allow the maintenance of their spindle shape in the early stages, while at advanced stages the cell morphologies are less regular (large sizes and irregular geometries). The chondrogenic stimulation has been assessed with Alcian Blue staining, osteogenic with Alizarin staining, and adipogenic differentiation with Oil Red-O staining. In osteogenic differentiation, calcium oxalates formation could be observed on BMMSCs, but only on the differentiated ones. Oil Red-O permitted the staining of intracellular lipid droplets leading to BMMSCs adipogenesis. The sample that shows the lowest differentiates is the composite which contains NPs calcinated at 800 °C.

Conventional hydrogels are currently used for scaffold development, where they could provide physical and chemical biomimetic microenvironments for cell incorporation. However, these biomaterials have deficiencies related to dynamic changes and heterogeneity observed in native tissues [165]. As shown in Table 4, each application in tissue engineering provides advantages, and at the same time, limitations. The continuous research for their improvement must be further performed and demonstrated.

## 7. Remarks and Future Perspectives

Through this study, it can be concluded that polymeric scaffolds are ideal materials used in tissue engineering due to their ability to mimic the targeted tissue. In this regard, hydrogels can be optimized for each application. The high-water content, malleability, biocompatibility, and biodegradability make them suitable biomaterials for tissue engineering applications. This study describes the potential application of composite hydrogels and their main characteristics in the biomedical field. Also, both the source and the crosslinking method influence their physico-chemical and biological properties. Hence, the use of natural polymers allows the obtainment of hydrogels with low immunogenicity, high biodegradability, and superior biocompatibility, while their mechanical strength is still inferior. Unlike natural polymers, synthetic hydrogels have superior mechanical strength, but their biocompatibility and biodegradability capacities are inferior to natural polymers. To overcome the main drawbacks of natural and synthetic polymers, mixtures of natural and synthetic polymers have been developed to prolong the degradation time closer to the healing process. Another advantage is the improvement of mechanical properties and the ability to confer biocompatibility, even for the products resulting from degradation.

Moreover, introducing nanoparticles into the structure of hydrogels or various active principles (drugs, essential oils, or growth factors) can improve the scaffold’s biological properties when introduced into the body. Thus, we can see that the studies regarding the use of polymeric scaffolds in tissue engineering are continuously developing to optimize their properties with the ultimate goal of improving the healing process. Further, their use in applications such as controlled drug release is particularly important as the mechanism of active substances encapsulation and the release mechanism is particularly complex and thus requires special attention. Currently, the main challenge of hydrogels is represented by the need to create biomaterials with improved therapeutic effects. Their combination with nanoparticles and various active agents requires further optimization to avoid any cytotoxic effect and obtain the desired beneficial biological activity.

## Figures and Tables

**Table 1:** The main advantages and disadvantages of natural and synthetic polymers.

Polymer	Advantages	Drawbacks	References
Natural Hydrogels			
Alginate	Biodegradability, biocompatibility, proper for in situ injections, water-solubility, crosslinking under mild conditions	Mechanical weakness, difficulties in sterilization, handling, storage in solutions	[32,33,34,35]
Hyaluronic acid	Water-solubility, biocompatibility Biodegradability, low immunogenicity, promotes cell proliferation and differentiation, involved in wound healing phases	Mechanical weakness, high costs	[29,36,37,38,39]
Chitosan	Excellent host response, biodegradability, outstanding biocompatibility, antimicrobial activity, hydrophilic surface, provides cell proliferation, adhesion, and differentiation	Mechanical weakness, extremely viscous, soluble in acidic solutions, expensive purification	[40,41,42,43]
Gelatin	Water-solubility, obtained from different animal by-products, Forms high mechanical and thermo-revisable hydrogels, Forms easily matrix hydrogels and films	Extremely viscous, quick biodegradation, inferior thermal stability at increased temperatures	[44,45,46,47]
**Synthetic Hydrogels**			
Poly (N-isopropyl acrylamide) PNIPAAm	Soluble in water, temperature-responsive polymer, superior mechanical properties, biocompatible, used for controlled drug delivery and tissue engineering	Requests chemical crosslinking, cytotoxicity, poor thermal stability	[48,49,50,51,52]
Polyethylene glycol (PEG)	Good mechanical properties, low toxicity, reproducible synthesis, soluble in water	Poor cell affinity, decreased cellular response, low cell adhesion	[53,54,55,56]
Poly (vinyl) alcohol (PVA)	Soluble in water, non-toxic, good mechanical properties, film-forming ability, biocompatible	Does not support cell proliferation and attachment, limited hydrophilicity, insufficient elasticity	[57,58,59,60,61]

**Table 2:** Scaffolds based on hydrogels applied in tissue engineering and drug delivery.

Materials	Active Agents	Properties	Applications	References
cellulose	methoxy pectin	favorable rheological properties, tissue compatibility, water absorption	3D printing	[118]
silk fibroin	gelatin	excellent structural stability, increased biocompatibility, cell fixation, and proliferation	3D printing	[119]
hydroxyethyl cellulose	silver nanoparticles	improved mechanical properties, antibacterial properties, green and simple strategy for Ag NPs, biocompatibility	antibacterial strain sensor	[120]
carbopol	wax gourd extract and capsicum extract nanoparticles	reduced cytotoxicity, enhanced permeation, controlled release,	transdermal delivery	[121]
silk sericin	Fe_3_O_2_ NPs, secretome	reduced toxicity compared to other delivery systems for cardiomyocytes	injectable carrier for ultrasound contrast agents	[122]
gelatin/oxidized alginate	nanohydroxyapatite	improved rheological and mechanical properties, cytocompatibility,	bone tissue engineering	[123]
polyacrylamide/N-methylenebisacrylamide	silver nanoparticles	increased mechanical properties, excellent antimicrobial activity	wound dressings	[124]
hydroxypropyl methylcellulose	Cu NPs	size-dependent antibacterial activity	antibacterial applications	[125]
modified platelet lysates	dexamethasone loaded mesoporous silica NPs, bone marrow-derived mesenchymal stem cells	the bioactive content which modulates cell fate, cell differentiation, suitable biochemical microenvironment, increased biocompatibility	bone regeneration and repair	[126]
oxidized alginate, carboxymethyl chitosan	hydroxyapatite	self-healing property, high porosity, increased cytocompatibility, tunable gelling features	injectable hydrogels for bone tissue engineering	[127]

**Table 3:** Hydrogel based formulations used for skin tissue engineering as clinical trials.

Clinical Trial Model	Formulation	Polymers	Active Agent	Teste Bacteria	References
Mice	Dressing	Alginate	CM11 peptide	MRSA	[144]
	Gel	Cellulose (Hydroxypropyl cellulose)	PXL150 peptide	*P. aeruginosa*	[145]
	Dressing	Chitosan acetate	Silver nanoparticles	*A. baumannii;* MRSA; *P. mirabilis; P. aeruginosa*	[146]
	Hydrogel	Chitosan (glycol chitosan)/Aldehyde-modified poly(ethylene glycol) derivative	Colistin	*P. aeruginosa*	[147]
	Hydrogel	Hyaluronic acid/Dextran	Sanguinarine (loaded in gelatin microspheres)	*E. coli;* MRSA	[148]
	Hydrogel	Hyaluronic acid	Sanguinarine (loaded in gelatin microspheres)	*E. coli; S. aureus*	[149]
Rats	Hydrogel	Alginate	Honey	*A. baumannii; K. pneumoniae;* *P. aeruginosa; S. aureus*	[141]
	Film	Cellulose (Sodium carboxymethyl cellulose)	-	*P. aeruginosa; S. aureus*	[150]
	Scaffolds	Cellulose/Collagen	Curcumin (loaded in the gel)	*E. coli; P. aeruginosa; S. aureus*	[151]
	Dressing	Chitosan acetate	-	*P. mirabilis; P. aeruginosa; S. aureus*	[147]
Rabbits	Hydrogel	Chitosan/Collagen	Lysostaphin	MRSA	[147]
	Nanoparticles	Chitosan (Carboxymethyl chitosan)	-	*P. aeruginosa; S. aureus*	[152]
Human	Dressing	Alginate	Silver	-	[153,154]
	Topical spray	Hyaluronic acid	Metallic silver	-	[155]
	Dressing	Cellulose	-	-	[156]
	Hydrogel	Chitosan/Dextran	-	-	[157]

**Table 4:** The main advantages and limitations of hydrogels applied in tissue engineering.

Application	Advantages	Disadvantages	Future Perspectives	Reference
Skin	Controlled biodegradation rate, increased biocompatibility, promote wound healing, high swelling ability.	Decreased mechanical strength due to soft structures.	Degradation behavior and tenability should be further studied. Hydrogels incorporating growth factors (GF) could facilitate cell differentiation.	[166,167]
Bone	Good biocompatibility, nonimmune response, control of cell-matrix interactions, adjustable properties through crosslinking.	Cell distribution cells within scaffolds may be restricted, with poor mechanical properties.	The addition of inorganic or organic/inorganic nanoparticles (NPs) ions may enhance the stiffness of the hydrogel, and change cells behavior or release speed of GF (e.g., transforming growth factor-β (TGF-β), bone morphogenetic protein (BMP), fibroblast growth factor, (FGF) or insulin-like growth factor (IGF)) The organic-inorganic hybridization can be an efficient strategy to synthesize smart hydrogels.	[168,169,170]
Cartilage	Adjustable physicochemical properties, versatility, biocompatibility, and high similarity to the natural ECM.	When subjected to cyclic stress, hydrogel bonding can break due to a lack of mechanical integrity.	The addition of nanoparticles, organic/polymeric composites, and inorganic agents (such as clay, hydroxyapatite, metallic nanoparticles, or graphene) can be used as fillers to reinforce the scaffold	[171,172,173,174]

## Data Availability

The data presented in this study are available on request from the corresponding author.
